# Improved capture of *Drosophila suzukii* by a trap baited with two attractants in the same device

**DOI:** 10.1371/journal.pone.0188350

**Published:** 2017-11-17

**Authors:** Rodrigo Lasa, Eduardo Tadeo, Ricardo A. Toledo-Hérnandez, Lino Carmona, Itzel Lima, Trevor Williams

**Affiliations:** 1 Red de Manejo Biorracional de Plagas y Vectores, Instituto de Ecología AC, Xalapa, Veracruz, Mexico; 2 Departamento de Investigación Aplicada, Driscoll’s, Zapopan, Jalisco, Mexico; Institut Sophia Agrobiotech, FRANCE

## Abstract

The improvement of trap-lure combinations is an important part of integrated pest management programs that involve monitoring pests for timely insecticide applications, or for their use in control strategies such as mass trapping or bait stations. In this study improvements in the capture of *Drosophila suzukii* were not observed following the inclusion of different color stimuli with respect to a red-black stripe cup trap. This red-black stripe trap with a hemispherical dome-shaped lid had a significantly improved physical retention of flies compared to traps fitted with a flat lid. Retention was further improved when an additional tube device, which could be baited with a supplemental attractant, was introduced through the dome-shaped lid. Under laboratory conditions, this trap, in which apple cider vinegar + 10% ethanol was present as the drowning solution and the additional tube device was baited with a fermenting mixture of sugar and yeast, was significantly more effective in catching *D*. *suzukii* flies than other conventional attractants or a commercial lure. The capture rate of this trap-lure combination remained higher than that of a commercial lure, even after 20 days of use under laboratory conditions. In a guava orchard this trap was 15-fold more effective in catching *D*. *suzukii* flies than similar traps baited with apple cider vinegar alone, 4 to 7 fold more effective than similar traps baited with a commercial lure, and 1.7-fold more effective than a fermenting mixture of yeasts and wheat flour. In commercial blackberry orchards, this trap was 6-fold more effective in trapping *D*. *suzukii* flies than the clear trap baited with apple cider vinegar used by growers. The efficacy of this trap presents a promising line of future research for monitoring and control of *D*. *suzukii* and likely other drosophilid pests.

## Introduction

The rapid spread of the invasive *Drosophila suzukii* (Matsumura) in different countries [1–4], and the important economic impact of this pest in soft fruits and other crops [5–7], has highlighted the need for effective systems for its detection and control [8–9]. Monitoring of insect populations is a key part of integrated pest management programs that allows timely application of control interventions aimed at minimizing pest-induced damage to crops [10]. Estimates of *D*. *suzukii* populations can be achieved by active sampling of larvae in fruits [11], although trapping adults with targeted trap and lure combinations is a cost-effective approach that can be applied over large areas, and is markedly less labor intensive than manual sampling of infested fruit. However, efficient monitoring programs require effective traps with high attraction and good levels of selectivity to the pest(s) of interest [12, 13].

Driven by the need to improve systems for monitoring invasive *D*. *suzukii* populations, researchers have been testing the efficacy of a variety of commercial and handmade trap prototypes [13–16]. Several physical features such as trap color, size, shape, upper or lateral entry holes, entry surface area, entry hole position, surface area of the bait-air interface, trap headspace and tent coverings, among others, have been studied in a diversity of trap models [13–18]. However, results have been variable due to the variety of modifications of different trap models, and their evaluation under different laboratory or crop conditions. This can hinder accurate comparisons of trap designs. As almost all of the features of a trap are inter-dependent, the modification of one feature can sometimes be highly influential on insect responses to other features, often in a specific manner for each kind of trap model. Moreover, biotic and abiotic factors, such as the attractant used, type crop, insect maturity, trap position, distance between traps, contrast background and weather conditions can strongly influence fly captures which can add a further layer of complexity to comparisons of trap designs [13–16]. Despite all these differences, the use of red-colored traps, sometimes with a black stripe visual stimulus, and with lateral entry holes, are proving to be more effective than the clear plastic cups that were used in the past [15, 16, 19, 20].

Moreover, researchers have been working steadily to improve the attractiveness of lures for this pest. Apple cider vinegar (ACV) has been commonly used because it is cheap, readily available and captured flies can be easily visualized due to its transparency [14], although studies have indicated that it may not be the most attractive lure available. Fermentation products such as acetic acid, ethanol and methanol have been used as lures for *D*. *suzukii* and several other drosophilids [21, 22]. Mixtures of wine and ACV were found to be more attractive than either substance alone [10]. Other fermentation products serve as attractants because yeasts are involved in courtship, egg production or as a food source for larval development [23]. As such, a significantly increased response of *D*. *suzukii* to fermenting lures has been observed when compared with the standard ACV attractant [18, 20, 22, 24].

This study aimed to evaluate a simple handmade trap that can be easily baited with two independent attractants. For this, handmade cup traps with different color stimuli were evaluated and compared to a previously tested red-black stripe cup trap. The use of a hemispherical dome-shaped lid was then evaluated in order to determine the physical retention of flies, a feature that reduces escape from the trap when comparing with a flat lid. Different combinations of lures were then tested for trapping efficacy under laboratory conditions. The most effective trap-lure combination was compared under field conditions, in a guava orchard, with other attractants commercially available or previously tested by other authors. Finally, this promising trap-lure combination was compared with the trap used by blackberry growers in Mexico under commercial greenhouse conditions.

## Material and methods

### Insects, traps and laboratory cages

A laboratory colony of *D*. *suzukii* was started in the insectary of the Instituto de Ecología AC, Xalapa, Veracruz State, Mexico, using pupae that emerged from guava fruits, *Psidium guajava* L., collected from orchards adjacent to the town of Xico, Veracruz State, in September 2015. Flies from the colony were allowed to oviposit in a cornmeal-based artificial diet [25], dispensed into 300 ml plastic cups and covered with fine nylon gauze. The colony was maintained at 24 ± 1°C, 60 ± 10% relative humidity (RH) and 12:12 h (L:D) photoperiod with a 10 W led light (Megamex, Mexico City) intensity of 3500–4500 lux. Male and female flies used in tests had been kept together in cages since emergence. Sexually mature, mated adult flies of 3 to 5 days old were used for laboratory observations.

**Fig 1:**
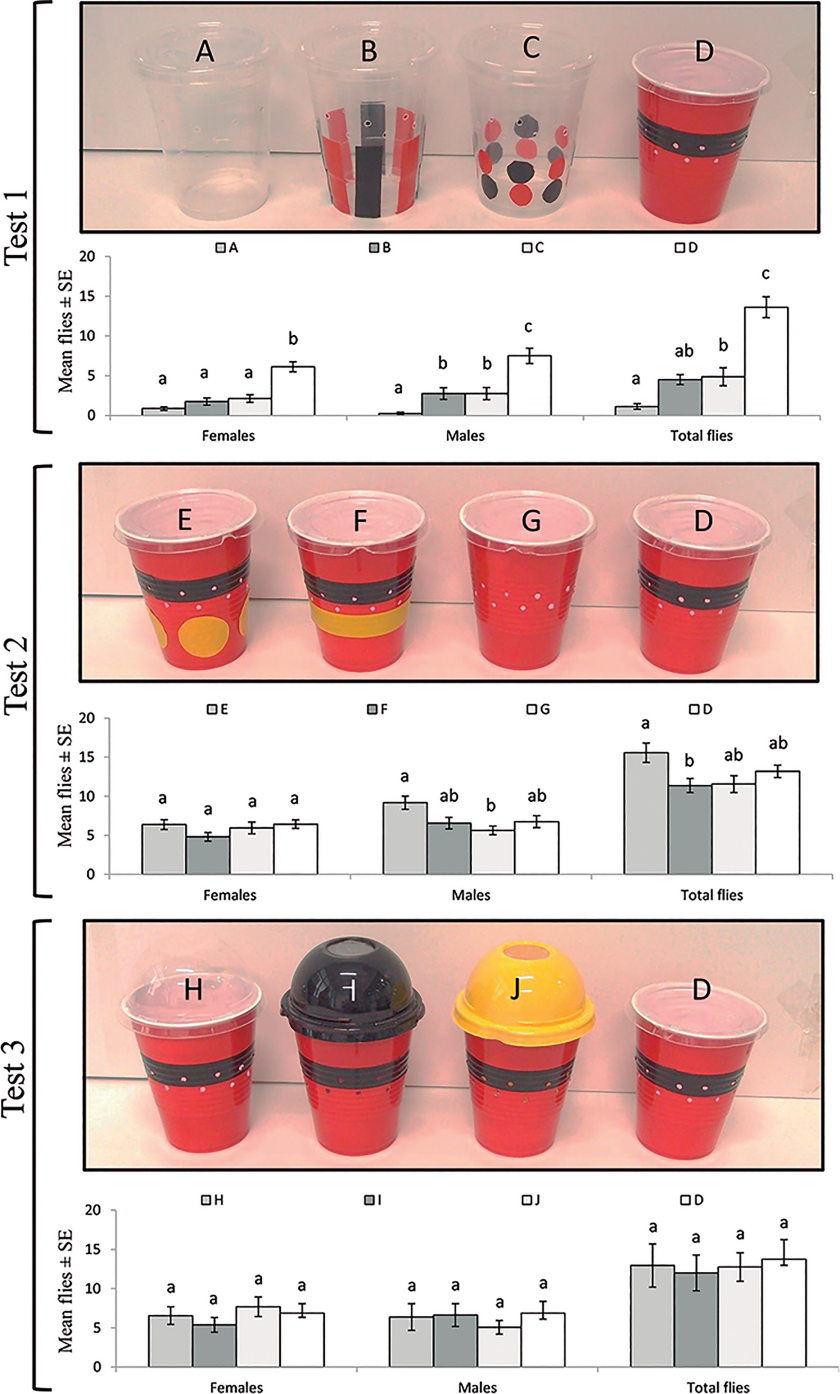
Efficacy of different trap models of trap tested against *Drosophila suzukii* flies in three independent tests developed under laboratory cage conditions. The red-black stripe trap D was used as the reference trap in all tests. Test 1: stimuli of small circular or vertical red-black bands in colorless cup traps A-D; Test 2: inclusion of yellow circles or bands on red-black stripe traps E-D; Test 3: contrasting color effects using transparent or colored dome shaped lids that improved headspace between the access holes in red-black stripe traps H-D. Columns labelled with the same letter were not significantly different for comparisons of responses of each sex or total captures in each experiment (Tukey HSD test, P > 0.05).

**Fig 2:**
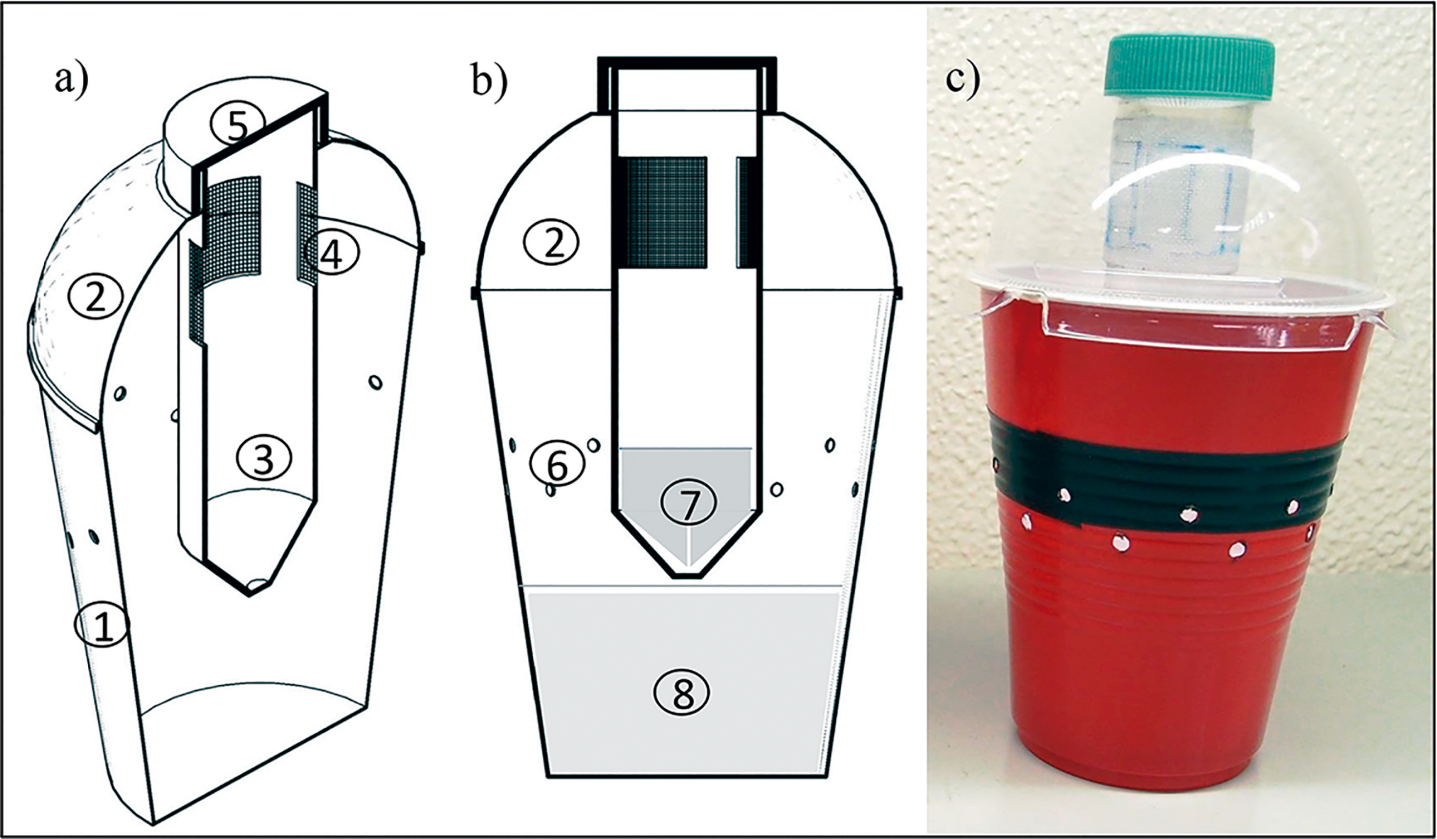
Trap model developed for use in experiments. (a) lateral section (b) frontal section of the trap design and (c) photograph of the trap. This trap was constructed with a red opaque cup and black horizontal stripe (1) fitted with a transparent dome-shaped lid (2) in which an additional tube device (3) was inserted through the upper opening of the dome lid. The cup was baited with an attractant drowning solution (8) and the additional device was baited with a complementary attractant (7), which was a sugar-yeast mixture in the present study. The additional tube device was closed with a plastic screw-top (5) that fitted precisely in the upper opening of the dome lid and prevented the escape of flies from inside the trap. Volatiles from the additional device passed through a nylon mesh 0.2 mm (4), mixed with drowning solution volatiles in the internal cup space and were released through lateral access holes (6). This model trap was called “2C-Trap” when baited with ACV + 10% ethanol as the drowning solution and with a sugar- yeast mixture in the additional tube device.

Traps were constructed using opaque red polyethylene cups (470 ml; Great Value, Wal-Mart de México S de RL de CV, Santa Cruz Acayucan, Mexico) modified with 20 holes of 3.2 mm diameter in two rows (10 equidistant holes per row) in the upper middle of the cup (Fig 1). The size of the holes was based on the size of access holes used in previous traps [14]. Some cups had a stripe of black electrical tape at the height of the upper row of holes. Holes were homogeneously distributed around the cup. Two transparent lid models (Reyma, Ecatepec, Morelos) were used: a hemispherical dome-shaped lid (Fig 1H) and a flat lid (Fig 1D). The hemispherical dome-shaped lid had a circular hole in the upper part that was closed with a transparent plastic circle (Fig 1H–1J), or was left open and used to introduce an additional tube device that was baited with an additional attractant (Fig 2). The additional device inserted in the trap was constructed using a 50 ml polyethylene centrifuge tube in which three 4 cm^2^ rectangular openings were perforated 1 cm below the screw-cap (Fig 2B). These rectangular sections were covered with 0.2 mm nylon mesh to allow volatiles to escape into the trap headspace and to prevent flies entering the tube device. In this case, the attractant used to capture flies and the attractant included inside the additional tube device were not in contact, but volatiles from both sources could mix in the trap headspace.

To compare our trap-lure design, a colorless transparent 0.47 l cup (Plásticos Adheribles del Bajío, León, Mexico) with 20 lateral holes 3.2 mm was also used for laboratory and guava tests (Fig 1A). A clear 1 l capacity plastic cup with a flat lid (Plásticos Adheribles del Bajío, León, Mexico) with 10 holes of 4 mm diam. was used in blackberry greenhouse tests, as this was the trap model currently being used by berry growers in Mexico. These colorless cup traps are currently recommended for monitoring *D*. *suzukii* populations by the Ministry of Agriculture, Rural Development, Fisheries and Food (SAGARPA) and the National Service for Agroalimentary Public Health, Safety and Quality (SENASICA) in Mexico [26]. We refer to these as the SAGARPA-recommended traps.

Visual stimuli involving red, black and yellow colored circles and bands on the outside of cups were made using colored paper with adhesive on one side (Lustrin-Ikw^®^, Mexico). Dome-shaped lids were painted using yellow, red and black acrylic paint (Comercial Mexicana de Pinturas SA de CV, Tepexpan, Mexico) (see Fig 1I and 1J).

The attractants used in experiments were prepared using (i) apple cider vinegar (ACV, 5% acetic acid) (La Costeña, Ecatepec, Mexico), or (ii) the commercial lure SuzukiiTrap^®^ (Bioibérica, Barcelona, Spain), consisting of a mixture of organic acids and peptides, and (iii) a yeast-sugar mixture of 20 ml of water, 1.1 g sugar and 0.417 g of yeast. Dry active baker's yeast (*Saccharomyces cerevisiae*) (Tredi-Pan, Safmex SA de CV, Mexico-Toluca, Mexico) was used for all tests.

### Cage experiments

#### Effect of trap color stimuli on fly attraction

To determine the effect of various color stimuli on the capture efficacy of *D*. *suzukii* three consecutive tests were performed using a similar methodology under caged conditions in the laboratory. Sets of four traps were evaluated in three independent tests. In all tests the red-black stripe trap design was included as a reference (Fig 1D). In test 1, the stimulus of small circular or vertical red-black bands (similar in size to blackberry fruits) on transparent cups; test 2 (Fig 1E–1D) to determine a possible improvement of attraction with the inclusion of yellow circles or bands in a red-black stripe trap. This yellow stimulus is related with high presence of this pest infesting guava in our region [26]; and test 3 (Fig 1H–1D) to determine contrast color effect using transparent or colored dome shaped lids that improve headspace between the access holes and the lid, a feature that previously improve trap capture [18]. For all these tests, the red-black stripe trap with a transparent flat lid (Fig 1D) was used as the reference trap because it had been recommended for berry growers as a *D*. *suzukii* monitoring trap by Cornell University [19], based on promising results published elsewhere [15]. Each set of traps was evaluated simultaneously using cages of PVC tubular frame (0.6 x 0.6 x 0.9 m) covered with a white 1 mm nylon mesh. These experimental cages had four equidistant elastic lines placed across the top of each cage that were used to hang galvanized wires, from which traps were suspended at a height of 65 cm above the base of the cage. A single blackberry plant, *Rubus fruticosus* L., without fruits, 80 cm high and planted in a plastic pot with soil, was placed inside each cage as the resting site for adult flies. Sets of four traps were baited with 50 ml ACV containing 10 μl of Tween 80 to reduce the surface tension of the liquid. A single trap was hung at each of the four corners of the cage. Within each cage, 40 adult females and 40 adult males of *D*. *suzukii* (3 and 5 days old, respectively, and not starved), were gently released onto the blackberry plant using an entomological aspirator. Female flies of this age actively search for an oviposition substrate [27]. Laboratory conditions were 26 ± 1°C, 60 ± 10% relative humidity (RH) and 16:8 h (L:D) photoperiod. Twenty-four hours after flies were released, the total numbers of females and males captured in each trap model were evaluated. Two identical cages were prepared for test 1 (Fig 1A–1D) and a total of four replicates were performed by changing the position of the traps sequentially so that each trap was tested for a 24 h period in each corner of the cage. For tests 2 and 3, four identical cages were prepared with a similar methodology; the position of the traps was changed sequentially so that each trap was tested for a 24 h period in each corner of the cage.

#### Influence of trap lid shape on the physical retention of flies

The physical retention capacity of cup traps, referred to the ability of the trap to retain adult flies once they have entered the trap, was compared using a dome shaped transparent lid and a flat transparent lid under laboratory conditions in two independent tests. In a first experiment, two transparent cup trap models were compared, one with a flat lid (Fig 1A) and one with a dome lid (Fig 1, cup of trap A with lid of trap H). A single cup trap was placed inside a 30x30x30 cm Plexiglas cage with 0.1 mm nylon mesh in order to contain flies that escaped from the trap. A group of 24 *D*. *suzukii* laboratory-reared flies (12 males and 12 females aged 3 days and not starved) were gently released inside each trap using an entomological aspirator and were observed for a period of 30 min. The number of flies that managed to escape from the trap in 30 min was counted and classified by sex. All traps were evaluated in the absence of an attractant and without insecticide or other retention system. A total of 20 replicates of each trap lid design were performed for this experiment. Experiments were performed under laboratory conditions at 24 ± 1°C, 60 ± 10% RH.

In a second experiment, the physical retention of a red-black stripe trap with a flat lid (Fig 1D) was compared with that of the same opaque red-black stripe cup with a dome lid that had the additional tube device inserted in the lid (Fig 2). Following the methods described in the first experiment, 12 adult females and 12 adult males were released into each trap and the number of flies that managed to escape from the trap in 30 min, was counted and classified by sex. A total of 10 replicates were performed for this second experiment.

#### Attraction to trap-lure combinations under laboratory conditions

**Fig 3:**
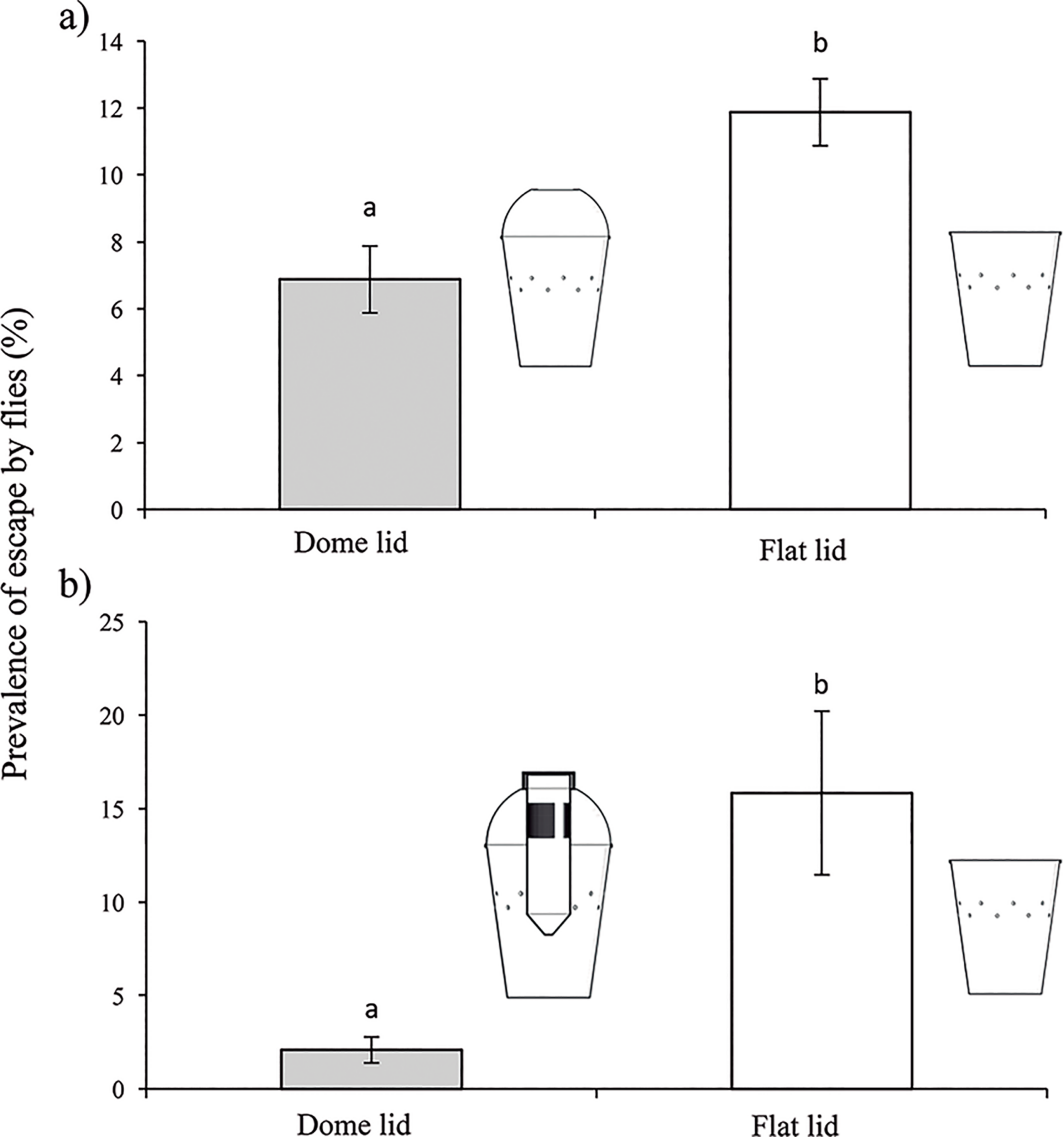
Mean (± SE) percentage of flies that escaped from different trap models in 30 minutes. (a) comparison between dome-shaped and flat lids in a colorless transparent cup trap and (b) comparison between red colored cup trap with a dome lid and an additional tube device and a similar red cup trap with a flat lid. As flies were initially placed inside traps, the observations were performed using traps without lures.

The capture efficacy of two sets of trap-lure combinations was evaluated under laboratory caged conditions using the red-black stripe trap with the hemispherical lid that was identified as the most effective combination in previous tests (Figs 1H and 3).

Initially, four trap-lure combinations were evaluated: (i) the red-black stripe trap baited with 50 ml ACV as a drowning solution in the bottom of the cup and the additional tube device (Fig 2) baited with a mixture of 20 ml of water, 1.1 g sugar and 0.417 g of yeast, subsequently referred to as the sugar-yeast mixture. This mixture was prepared immediately prior to use. (ii) The red-black stripe trap with the hemispherical dome shaped lid but without the tube device (Fig 1H) baited with 50 ml of a mixture of ACV + 10% ethanol (comprising 45 ml ACV + 5 ml ethanol). (iii) The red-black stripe trap baited with 50 ml of water in the bottom and the additional tube device with sugar-yeast mixture described in (i) (Fig 2), (iv) The red-black stripe trap without the additional tube device (Fig 1H) baited with 50 ml ACV alone. This trap lure combination was considered as the reference trap. In all cases, the retention liquid, either ACV or water, contained 10 μl of Tween 80 to reduce the surface tension of the liquid. Trap-lure combinations were evaluated simultaneously using cages (0.6 x 0.6 x 0.9 m) containing a single potted blackberry plant placed at the center, following the methodology described in the color stimulus experiments. The capture of the four trap-lure combinations was evaluated 24 h after 40 adult females and 40 adult males of *D*. *suzukii* (3 and 5 days old and not starved) had been released into the cage. Four independent cages were prepared simultaneously. The four trap-lure combinations were placed randomly in each corner of the cage. A total of four replicates were performed for each cage (n = 16) by changing the position of the trap at 24 h intervals.

**Fig 4:**
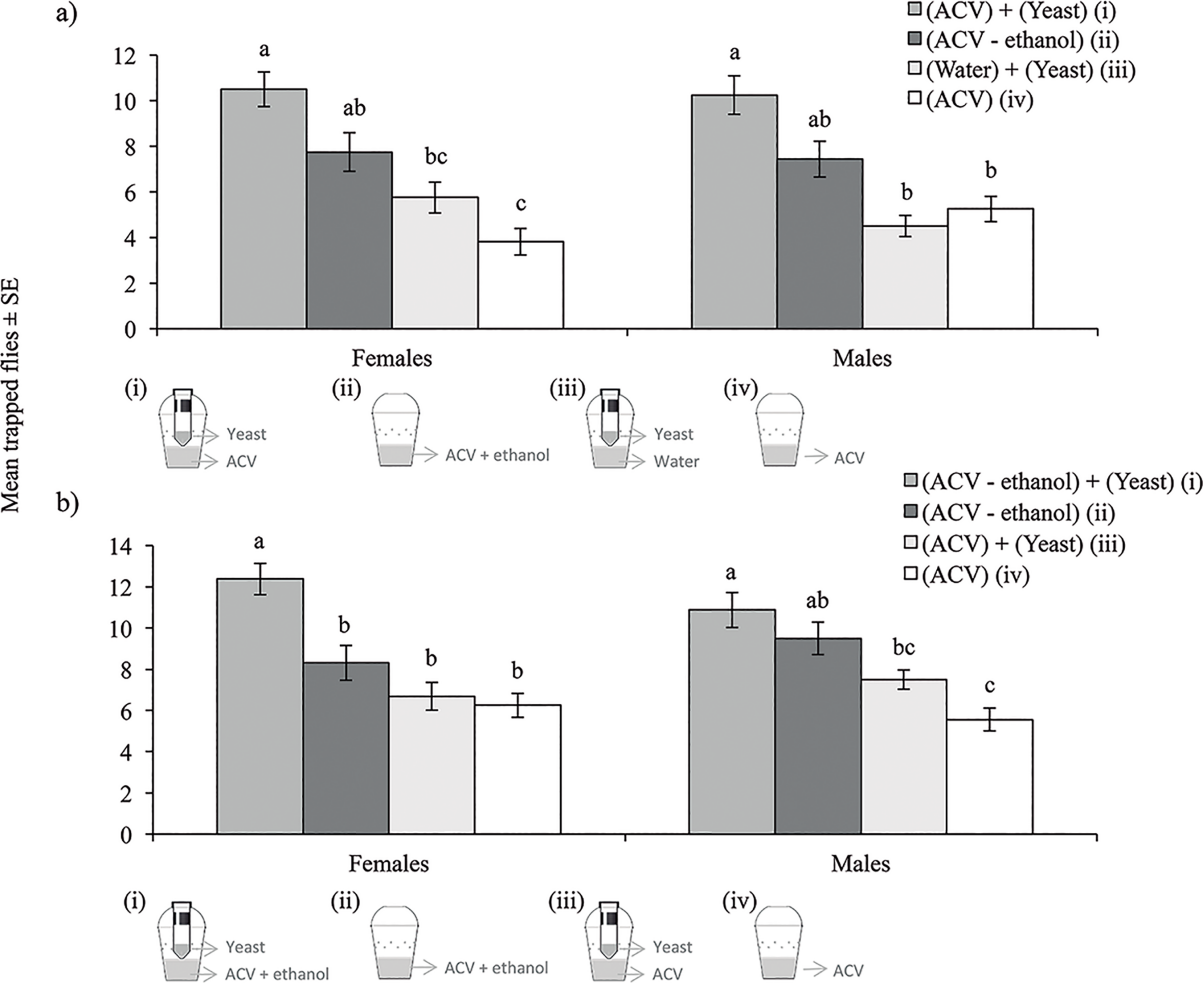
**Mean (± SE) numbers of *Drosophila suzukii* females and males trapped in two independent experiments (a, b).** Groups of 40 male and 40 female adults were released under cage conditions. Bars labelled with the same letter were not significantly different for comparisons of responses of each sex in each experiment (Tukey HSD test, P > 0.05).

Following favorable results for the ACV + ethanol mixture in the previous experiment (Fig 4A), a second test was performed with a similar methodology using the following four treatments: (i) the red-black stripe trap baited with 50 ml ACV + 10% ethanol as the drowning solution in the bottom of the cup and the additional tube device baited with the sugar-yeast mixture (Fig 2), (ii) the red-black stripe trap without the additional tube device (Fig 1H) baited with 50 ml of ACV + 10% ethanol, (iii) the red-black stripe trap baited with 50 ml of ACV as the drowning solution and the additional tube device baited with the sugar-yeast mixture (Fig 2) and, (iv) the red-black stripe trap without the additional device (Fig 1H) baited with 50 ml of ACV, and considered as the reference trap. In all cases the retention liquid contained 10 μl of Tween 80 to reduce the surface tension. Both tests were performed under laboratory conditions at 26 ± 1°C, 60 ± 10% RH and 12:12 h (L:D) photoperiod.

#### Efficacy of 2C-Trap *vs*. a commercial lure under laboratory conditions

The most effective trap-lure combination in capturing *D*. *suzukii* in the previous experiments is shown in Fig 4B. This trap was named "2C-Trap" and was compared with the effectiveness of a commercial lure using cages (0.6 x 0.6 x 0.9 m) containing a single potted blackberry plant. Commercial lure, SuzukiiTrap^®^, was selected for comparison because has been reported to be more attractive to *D*. *suzukii* than ACV in mixtures with sugar and yeast when tested in berry crops [18, 24, 28]. The two attractants compared were: (i) the red-black stripe trap (Fig 2) baited with 50 ml of ACV + 10% ethanol (including 10 μl of Tween 80) and the additional tube device containing freshly prepared sugar-yeast mixture (2C-Trap), and ii) the red-black stripe trap without the additional device (Fig 1H) with 50 ml of SuzukiiTrap^®^. Within each cage, traps were randomly assigned to two opposite corners of the cage with two traps per cage. The position of traps was alternated in each cage at 24 h intervals. A total of 40 adult females and 40 adult males of *D*. *suzukii* (3 and 5 days old and not starved), were released in each cage. Twenty-four hours after release, the total number of females and males captured in each trap model was recorded. A total of 4 replicate cages were used simultaneously with each type of trap placed twice at each position in each cage (n = 16 replicate observations).

Both lures were also evaluated in a subsequent test. The effectiveness over time of lures was compared under laboratory conditions for 20 days without renewing either of the attractants. For the test, the same trap-lure combinations described in the previous experiment were evaluated simultaneously with 4 cages, each containing a potted blackberry plant, and with traps randomly assigned to two opposite corners of the cage. The capture effectiveness was evaluated at day 0 (the day of preparation of the lures) and at 3, 6, 9, 12, 15 and 20 days after the start of the trial. For each evaluation day, 40 adult females and 40 adult males of *D*. *suzukii* (3 to 5 days old and not starved), were released in each cage. Twenty-four hours after release, the total number of females and males captured in each trap model was recorded. Trapped flies were sexed and discarded. The percentage of flies caught in each trap-lure combination (n = 4 replicates/time point), based on the total number of flies trapped in each cage, was averaged for each evaluation time during the trial. Both tests were performed under laboratory conditions at 26 ± 1°C, 60 ± 10% RH and 12:12 h (L:D) photoperiod.

### Field experiments

#### Attraction of 2C-Trap vs. other trap-lure combination in a guava orchard

A first field experiment where conducted during 8 days in September 2016 in a row of guava trees commonly infested by *D*. *suzukii* [26] located on the outskirts of the town of Xico, Veracruz state, Mexico (19°25'59.92" N; 97°1'58.88" W, 1385 m altitude). Our 2C-Trap (Figs 2C and 4B) was compared with SuzukiiTrap^®^ under field conditions. The comparison was performed using two treatments: i) the red-black stripe trap baited with 150 ml of ACV + 10% ethanol (including 10 μl of Tween 80) and the tube device containing a sugar-yeast mixture (2C-Trap), and ii) the red-black stripe trap without the additional device baited with150 ml of SuzukiiTrap^®^. A volume of 150 ml was used in field tests to reduce the effect of lure evaporation. Two traps of each treatment were randomly placed on guava trees in four different blocks along the row line. Traps were placed at 3 m height, 6–10 m apart, with a distance of 10–15 m between blocks. The traps were rotated once after 4 days so that traps were in both positions during the test.

In a second experiment, the effectiveness of the red-black stripe trap with ACV + ethanol and yeast-sugar mixture was compared with other trap-lure combinations in the same guava orchard during September-October 2016. The trap-lure combinations evaluated were: i) the red-black stripe trap baited with 150 ml of ACV + 10% ethanol mixture and the additional tube device with a sugar-yeast mixture (2C-Trap), ii) the red-black stripe trap without the additional device (Fig 1H) baited with 150 ml ACV + 10% ethanol, and iii) the trap model recommended by SAGARPA, consisting of a transparent plastic cup of 470 ml capacity with a transparent flat lid (Fig 1A), containing 150 ml of ACV. All attractants contained 10 μl Tween 80 to reduce surface tension. Four traps, one of each trap-lure combination, were placed randomly in four different blocks. The traps were placed 3 m high on tree branches at a distance of 6–7 m between traps in four different blocks 10–15 m apart. The traps were checked at 4 day intervals for 12 days. Captured flies were placed in 70% ethanol and taken to the laboratory for identification and counting. At each sampling time traps were rotated sequentially by position so that all the traps were placed once at each position within a block. During the experiment, 4 traps fell to the ground and could not be counted.

A third experiment was performed in the same guava orchard at the beginning of November 2016 to evaluate the efficacy of our best trap-lure combination compared to a fermenting lure previously described by Hampton et al. [20]. Two treatments were evaluated: i) the red-black stripe trap baited with 150 ml of ACV + 10% ethanol mixture and the additional tube device with a freshly prepared sugar-yeast mixture (2C-Trap), ii) the red-black stripe trap without the additional device (Fig 1H) baited with a mixture of 38 g wheat flour, 4.5 ml ACV and 2.6 g active yeast in 110 ml water, which also functioned as the drowning liquid [20]. Both attractants contained 10 μl of Tween 80. Two traps, one of each treatment, were randomly placed at 3 m height on guava trees in four different blocks. The traps were 6–10 m apart with a distance of 10–15 m between blocks. The traps were rotated every 3 days for 12 days (i.e., twice at each position). At each 3-day sample, captured flies were placed in 70% ethanol and taken to the laboratory for counting and *D*. *suzukii* identification.

For all experiments in guava, the total number of drosophilids captured in each trap-lure combination was counted. Adults of *D*. *suzukii* were sexed. The presence of another pestiferous drosophilid, *Zaprionus indianus* (Gupta), was also quantified but was not subjected to analysis. Numbers of trapped flies were transformed to flies per trap per day (FTD) prior to analysis.

#### Attraction of 2C-Trap vs. other trap-lure combination in a blackberry greenhouse

A final test was performed in commercial greenhouses, during May 2017, in two blackberry varieties in an orchard (~4 ha) placed in Jacona, Michoacan (Coord. 19°54'25.11"N, 102°12'15.85"W) following the interest of the berries growing sector to know the real differences on the capture efficacy between our improved trap-lure combinations and the trap they used based on recommendations by SAGARPA. Traps were evaluated independently in two commercial blackberry varieties (var. 082D60 and var. 086K2312) grown by the commercial producer Driscoll's in the region: i) the red-black stripe trap baited with 150 ml of ACV + 10% ethanol mixture (with 10 μl Tween 80) and the additional tube device with a freshly prepared sugar-yeast mixture (2C-Trap), ii) the trap recommended by SAGARPA, consisting of a transparent plastic cup of 1 l capacity with 10 holes of 4 mm, covered with a transparent flat lid and containing 150 ml of ACV. Each blackberry variety had been planted in one of two separate greenhouses in May 2015, were ~2.0 m in height and were in full fruit production during the tests. Two traps, one of each type, were randomly placed at a height of 1.7–1.8 m in four different blocks. Each block was taken as a row of plants. Traps were placed at least 10 m from the edge of the greenhouse. Traps within a block were placed 30 m apart and the treatments alternated between blocks. The traps were rotated every 7 days for 4 weeks (i.e., twice at each position). At each 7-day sample, captured flies were placed in 70% ethanol and taken to the laboratory. The total number of drosophilids captured in each trap-lure combination was counted and adults of *D*. *suzukii* identified and sexed. Numbers of trapped flies were transformed to flies per trap per day (FTD) prior to analysis. Both greenhouses were similarly managed and two pesticides were applied to blackberry crops during the test (S1 Table).

### Data analysis

As cage had no significant effect in any tests, the mean number of males, females and total flies caught in the different trap models was subjected to one-way analysis of variance. When comparing the two trap lid models, the percentage of flies that escaped each trap model was rank transformed to overcome issues of heteroscedasticity and compared by t-test. In trap-lure combinations evaluated under caged conditions, the numbers of trapped males and females were normalized by √(x + 0.5) transformation in test 1 and rank transformed to control heteroscedasticity in test 2. Transformed values were subjected to a two-way analysis of variance considering cage and treatment as factors. When comparing our red-black stripe trap baited with ACV + ethanol and the sugar-yeast mixture, against the commercial lure SuzukiiTrap^®^, the total numbers of flies trapped were normalized by √(x + 0.5) transformation and compared by t-test.

Blocks had no significant effects on captures in most tests performed under field (guava) or greenhouse (blackberry) conditions. Numbers of flies per trap per day (FTD) of *D*. *suzukii* and other drosophilids were √(x + 0.5) transformed, or rank-transformed if necessary, to control heteroscedasticity and compared by t-test. In the case of field test 2 performed in guava, FTD values of *D*. *suzukii* and other drosophilids were normalized by √(x + 0.5) transformation and subjected to two-way analysis of variance with block and trap model as factors. All analyses were performed using SPSS v.17 (SPSS Inc., Chicago, IL).

## Results

### Trap color stimulus on fly attraction

In the first test, significant differences were observed in the mean number of females (F = 24.27, df = 3, 28, P < 0.001), males (F = 18.26, df = 3, 28, P < 0.001), and total flies (F = 32.60, df = 3, 28, P <0.001) trapped in different trap models. The response to the red-black stripe trap (Fig 1D) was significantly higher than colorless traps or colorless traps with small red-black circles or bands stimuli (Fig 1, Test 1).

In the second test, the capture of females was similar for all traps (F = 1.43, df = 3, 60, P = 0.24), but significant differences were observed for males (F = 4.27, df = 3, 60, P = 0.008) and total flies (F = 3.66, df = 3, 60, P = 0.017). However, all traps had a similar or lower capture of flies than the red-black stripe trap that was used as the reference trap (Fig 2, Test 2).

In the third test, no significant differences were observed for the mean capture of females (F = 0.66, df = 3, 60, P = 0.580), males (F = 0.34, df = 3, 60, P = 0.800), and total flies (F = 0.09, df = 3, 60, P = 0.964) in traps with transparent and colored dome-shaped lids. The efficacy of the reference red-black stripe trap (Fig 1D) was similar that of the other traps (Fig 1, Test 3).

### Influence of trap lid shape on physical retention of flies

The colorless transparent cup trap with a dome shaped transparent lid was significantly more efficient in the physical retention of flies (Fig 3A) than the same cup with a flat transparent lid (t = 2.566; df = 38; P = 0.014). In this case, the prevalence of flies that escaped from the cup with the dome lid was approximately half that of flies that escaped from the flat lid trap. Similarly, the prevalence of escapes from a red-black stripe trap fitted with a dome lid containing the additional tube device (Fig 3B), was a small fraction of the flies that escaped from the same colored cup with a flat lid (t = 5.796, df = 18, P < 0.001). Based on these results we considered it important to use the dome-shaped lid in the following experiments.

### Attraction of trap-lure combinations under laboratory conditions

In the first experiment, significant differences were observed among trap-lure combinations for females (F = 10.02, df = 3, 48, P < 0.001), and males (F = 5.29; df = 3, 48, P = 0.003) (Fig 4A). The trap combining ACV in the base and containing the additional tube device baited with a sugar-yeast mixture captured approximately twice as many males and females of *D*. *suzukii* than ACV alone, or the same trap comprising the sugar-yeast mixture in the tube device with water in the base as the drowning solution. The capture of traps baited with ACV + 10% ethanol (without the sugar-yeast mixture) had intermediate values between both treatments (Fig 4A).

In the second experiment, the capture of different trap-lure combinations varied significantly for females (F = 14.21, df = 3, 48, P < 0.001) and males (F = 13.36; df = 3, 48, P < 0.001) (Fig 4B). The capture of females of *D*. *suzukii* observed in the trap combining ACV + ethanol with the additional tube device baited with a sugar-yeast mixture (2C-Trap) was significantly higher than in any of the other treatments (Fig 4B). Similarly, in the case of *D*. *suzukii* males, captures in the treatment involving ACV + ethanol with a sugar-yeast mixture in the tube device (2C-Trap) were significantly higher than all other treatments (Fig 4B), except that involving ACV + 10% ethanol (without the sugar-yeast mixture in the tube device).

### Efficacy of 2C-Trap vs. a commercial lure under laboratory conditions

**Fig 5:**
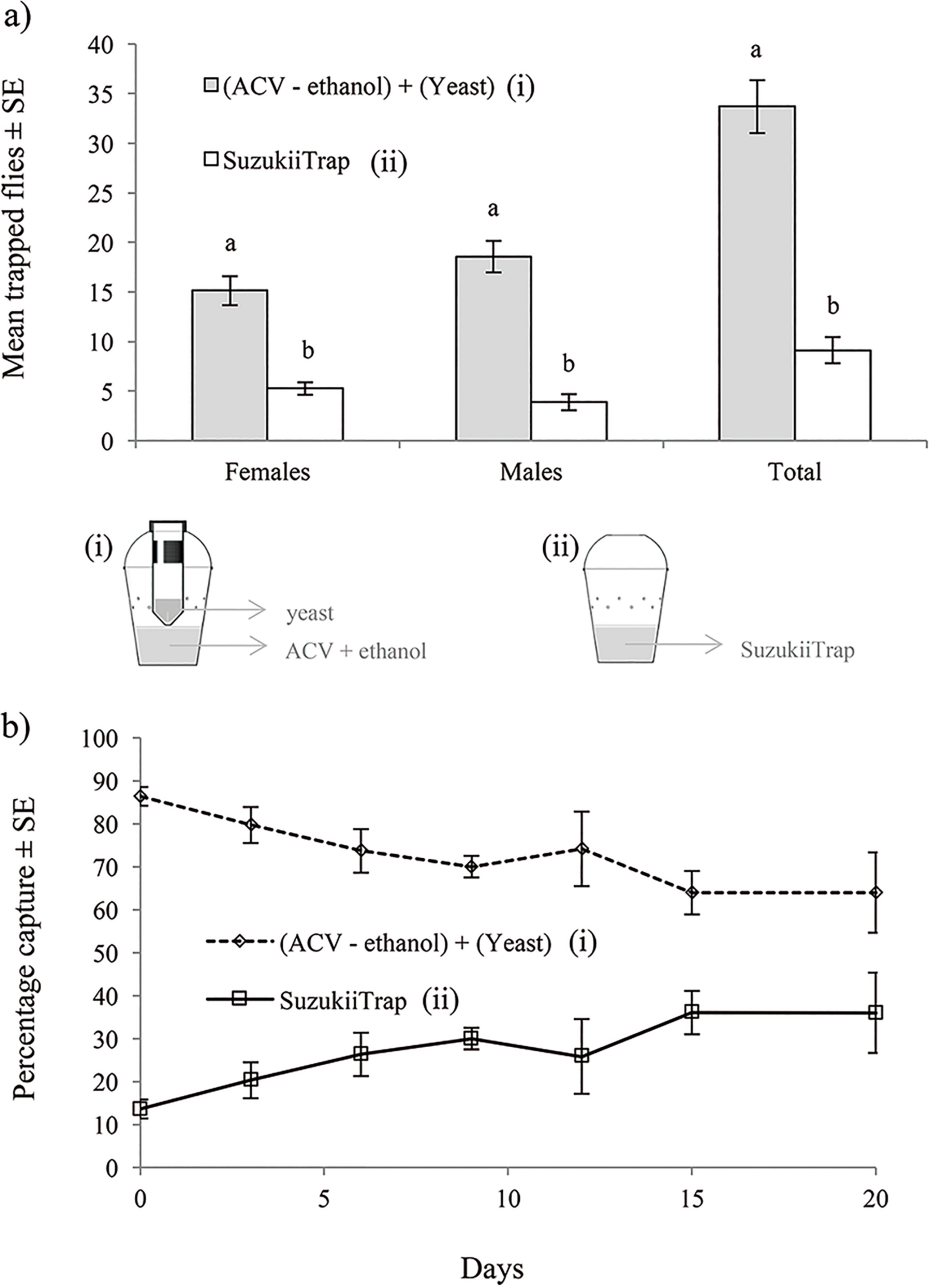
**(a) Mean (±SE) numbers of females, males and total flies (males + females) of *Drosophila suzukii*, trapped under cage conditions, in the 2C-Trap baited with (ACV + 10% ethanol) and sugar + yeast mixture, and the same trap with the commercial lure SuzukiiTrap; (b) mean (±SE) percentage of flies trapped in each trap-lure combination during a continuous test of 20 days under laboratory conditions.** Columns labeled with identical letters in (**a**) did not differ significantly for comparisons of trap captures within each sex (Tukey HSD, P > 0.05).

In the first experiment, our best trap that combined ACV + ethanol in the base and the additional tube device baited with a sugar-yeast mixture (2C-Trap), resulted in an approximately 3-fold higher capture of females (t = 6.99; df = 30, P < 0.001), males (t = 8.59; df = 30, P < 0.001) and total flies (both sexes) (t = 8.78; df = 30, P < 0.001), compared to a similar trap, baited with SuzukiiTrap^®^ (Fig 5A). Moreover, the efficacy for trapping *D*. *suzukii* of our trap trap-lure combination remained higher than that of the SuzukiiTrap^®^-baited trap for 20 days, despite the fact that lures were not renewed during the test (Fig 4B). However, the marked difference observed between these lures at the beginning of the test became gradually reduced during the course of the experiment (Fig 5B).

### Efficacy of 2C-Trap vs. other trap-lure combination in guava orchard

**Fig 6:**
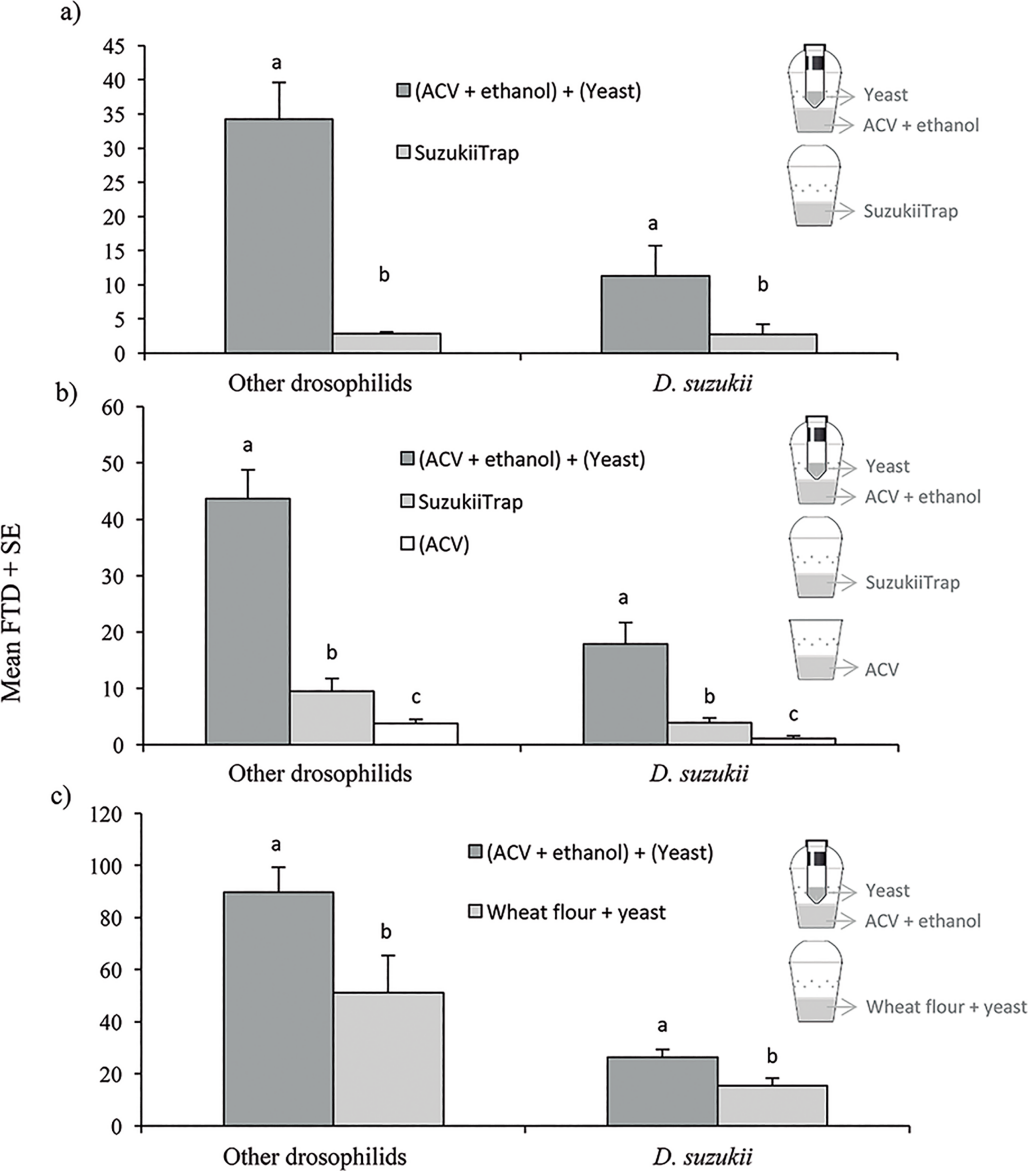
Mean (± SE) *Drosophila suzukii* flies and other drosophilids trapped in guava orchard trials using different trap-lure combinations in three independent field experiments. Different letters above columns indicate significant differences for comparisons of treatments within each class of insects in experiments 1 and 3 (t-test, P < 0.05) and experiment 2 (Tukey HSD, P < 0.05).

In the first field trial, a total of 2,173 drosophilid flies were trapped, of which 1986 individuals were *D*. *suzukii*, (59.8% females, 40.2% males). A significantly higher number of *D*. *suzukii* captures was observed in the 2C-Trap that combined ACV + ethanol and the tube device baited with a sugar-yeast mixture (t = 5.03, df = 14, P < 0.001), compared with SuzukiiTrap^®^ (Fig 6A). This 2C-Trap also captured significantly more drosophilids belonging to other species than the SuzukiiTrap^®^ baited trap (t = 5.41, df = 14, P < 0.001). Among these drosophilids, 284 were *Z*. *indianus* (56.7% females, 43.3% males). Indeed, almost all *Z*. *indianus* flies (95.7%) were trapped in the trap combining ACV + ethanol and the tube device containing sugar-yeast mixture.

In experiment 2, a total of 3,075 drosophilids were captured. Of these, 883 flies were *D*. *suzukii* (61.4% females, 36.6% males). Marked differences were observed in captures of *D*. *suzukii* (F = 48.83, df = 2, 20, P < 0.001) and other drosophilids (F = 129.99, df = 2, 20, P < 0.001) among trap-lure combinations, in which the 2C-Trap that combined ACV + ethanol and the tube device containing a sugar-yeast mixture captured approximately 4-fold to 7-fold higher numbers of *D*. *suzukii* and other drosophilids, respectively, compared to the SuzukiiTrap^®^ treatment (Fig 6B). The clear plastic trap baited with ACV alone, recommended by SAGARPA in Mexico, had the lowest capture of *D*. *suzukii* and other drosophilids. Among the other drosophilids captured, 776 flies corresponded to *Z*. *indianus* (74.6% females, 25.4% males). The majority of trapped *Z*. *indianus* (71.4%) were found in the novel trap combining ACV + ethanol and the tube device containing a sugar-yeast mixture.

In experiment 3, a total of 7,671 drosophilids were trapped during the 12 day trial. Of these, 1754 flies corresponded to *D*. *suzukii*, of which 33.6% were females and 66.4% were males. A significant higher number of flies per trap per day was observed for *D*. *suzukii* (t = 2.27; df = 26, P = 0.010) and other drosophilids (t = 2.83; df = 26, P = 0.009) in the 2C-Trap that combined ACV + ethanol and the tube device containing a sugar-yeast mixture, than in a similar trap without the additional device and baited with a lure comprising wheat flour, yeast and water (Fig 6C). Among the other drosophilids, 1105 flies corresponded to *Z*. *indianus* (53.1% females, 46.9% males). In this case, only 29.7% of trapped *Z*. *indianus* were captured in the 2C-Trap combining ACV + ethanol and the tube device with yeast suspension whereas 70.3% of *Z*. *indianus* adults were captured in the traps baited with the wheat flour and yeast lure described by Hampton et al. [20].

### Efficacy of 2C-Trap vs. other trap-lure combination in a blackberry greenhouse

**Fig 7:**
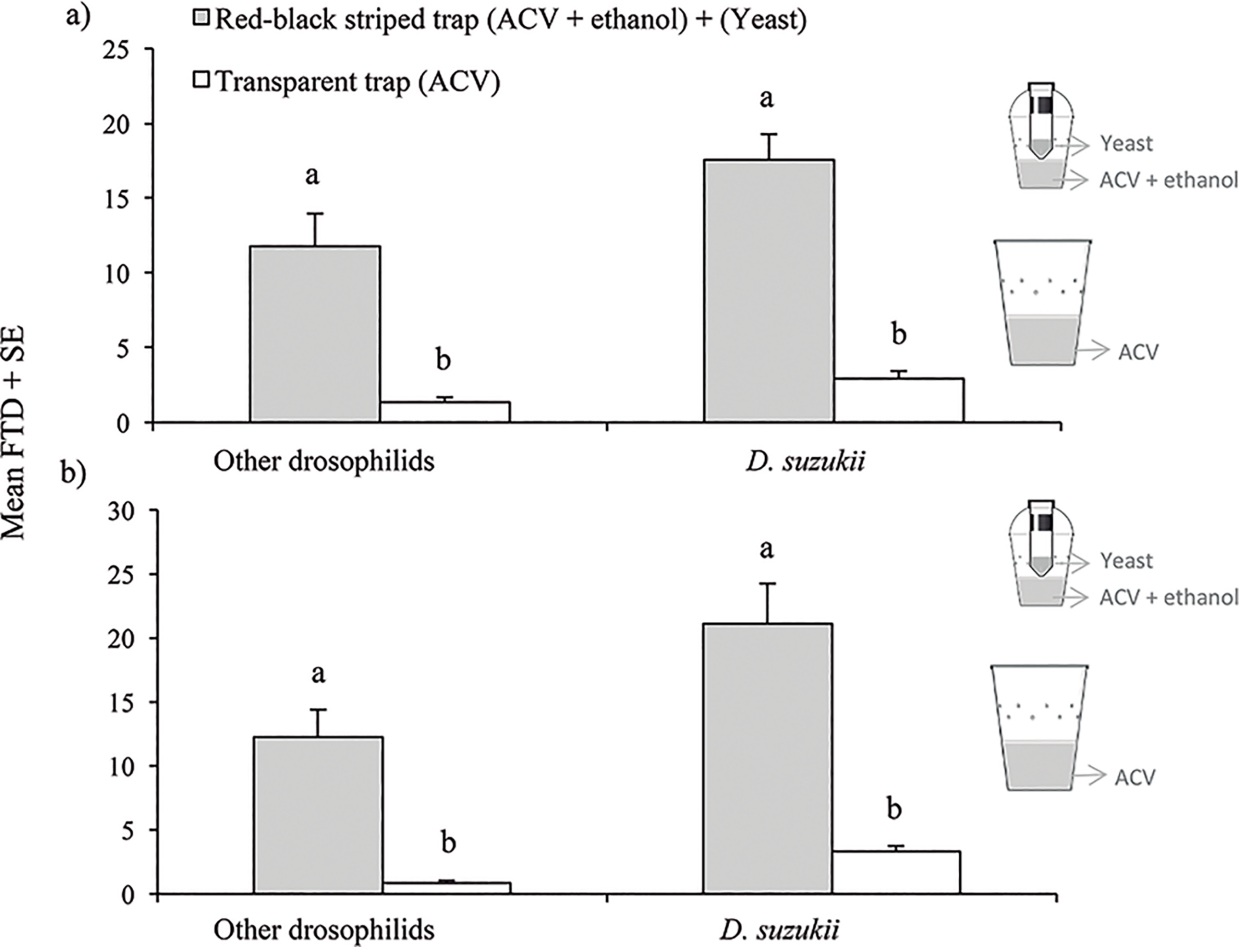
**Mean (± SE) *Drosophila suzukii* flies and other drosophilids trapped in two commercial greenhouses planted with different blackberry varieties using a red-black stripe trap with ACV + ethanol and a sugar-yeast mixture (2C-Trap) and the conventional clear plastic trap with ACV; (a) greenhouse planted with blackberry “var. 082D60” and (b) greenhouse planted with blackberry “var. 086K2312”**. Different letters above columns indicate significant differences for comparisons of treatments for *D*. *suzukii* and total drosophilids (t-test, P < 0.05).

A total of 7,968 drosophilids were trapped during 4 consecutive weeks in the trials carried out in two different blackberry varieties. Of these, 3,762 drosophilids were trapped in the greenhouse planted with blackberry “082D60” variety, of which 61.0% (2,249) were *D*. *suzukii* (61.0% females and 39.0% males). A significant 6 fold higher mean FTD value was observed for *D*. *suzukii* (t = 9.756; df = 30, P < 0.001) and other drosophilids (t = 6.951; df = 30, P < 0.001) in the red-black stripe trap baited with ACV + ethanol and the tube device containing a sugar-yeast mixture (2C-Trap), than in the standard 1 liter clear plastic trap baited with ACV (as recommended by SAGRAPA), that is used by berry growers in this region (Fig 7A).

A total of 4,206 drosophilids were trapped in the greenhouse planted with the blackberry “086K2312” variety of which 65.1% (2,739) corresponded to *D*. *suzukii* (67.5% females, 32.5% males). A significant 6.3 fold higher FTD value was observed for *D*. *suzukii* (t = 6.372; df = 30, P < 0.001) and other drosophilids (t = 5.265; df = 30, P < 0.001) in the red-black stripe trap that combined ACV + ethanol and the tube device containing a sugar-yeast mixture (2C-Trap), than in the standard 1 liter clear plastic trap baited with ACV (Fig 7B).

## Discussion

The present study demonstrated that the trap that comprised a perforated red opaque cup with a black horizontal stripe baited with a combination of ACV + 10% ethanol as the drowning solution (2C-Trap), and a fermenting sugar-yeast mixture in a ventilated tube device placed through the dome-shaped lid, was markedly more effective in capturing *D*. *suzukii* adults in laboratory and field conditions than a commercial lure (SuzukiiTrap) or a trap-lure combination recommended for capture of *D*. *suzukii* in Mexico by the Ministry of Agriculture, Rural Development, Fisheries and Food (SAGARPA) [26].

The cup design was based on the opaque red plastic cup (0.47 l) that was recommended by Carroll [19], and that contained features that were previously shown to improve capture efficacy, such as the red color [13, 15] and the stripe of black electrical tape placed close to the entry holes [15]. Two main modifications were made to this model in order to improve the efficiency and versatility of the trap: i) the use of a dome lid that favored fly retention and reduced the probability of flies escaping once they had entered the trap, and ii) the inclusion of the tube device in the dome lid that was baited with a complementary sugar-yeast attractant that can be replaced simply and quickly, without removing the trap from the field. The inclusion of the tube device allowed volatiles from two different attractants to become mixed inside the trap, without the need for the different components to be present in the same drowning solution. Furthermore, 20 access holes were homogeneously distributed around the upper middle of the trap. An outer sleeve was also constructed to slide over the lateral holes which allowed the trap to be emptied without spilling the drowning liquid and without the loss of drowned insects.

No modifications to the visual stimuli on the cup were necessary in field tests, because laboratory tests using different circles and bands stimulus of colors did not result in a significant improvement in fly captures when compared with the red-black stripe trap used as the reference model (Fig 1D) [19]. Although this pest infests guava, a yellow-colored fruit, in the central Veracruz region of Mexico [29], our previous observations were consistent with other findings in which yellow visual stimuli did not improve captures of *D*. *suzukii* when compared with red in several crops [14, 15, 22].

Interestingly, the use of a cup trap with a transparent dome-shaped lid improved the physical retention of flies that entered the trap in comparison with traps that used flat lids. The dome-shaped lid provides a larger light surface within the opaque cup and increases the volume between the access holes and the top of the trap, which allows flies to move upwards towards the light and away from the lateral entry holes, thus improving the probability of retention once flies have entered the trap. A similar effect was reported by Marcus [18], when the distance between trap entry holes and a flat lid was increased. Moreover, the introduction of an additional device appeared to increase fly retention (Fig 2), as flies flew directly from lateral access holes to the tube device located inside the trap, thereby reducing the time and distance walking over the cup wall, which likely resulted in a lower probability of finding an exit hole (R. Lasa, Personal observations).

Despite the improvement of fly retention in this model, the use of a combination of volatiles from ACV + 10% ethanol used as the drowning solution and an active fermenting sugar-yeast mixture in the tube device, markedly improve captures of *D*. *suzukii* flies compared with other attractants under laboratory and field conditions. The ACV + 10% ethanol attractant was used as the drowning solution because it was more efficient than traps baited with ACV alone. The ACV + ethanol mixture has a low surface tension, is transparent, and can be easily rinsed from captured specimens for subsequent species identification. In contrast, the dark color of SuzukiiTrap^®^ and the sticky characteristics of yeast-wheat flour attractants tend to hinder collection of trapped flies and their subsequent identification [22]. Carroll [19] recommended the use of an additional container that includes a fermenting attractant inside the bait drowning solution, but no efficacy data have been published on this combination with respect of other trap-lures. Moreover, in the case of this model trap, the inclusion of the device in the upper opening of the lid means that it can be changed quickly without opening the trap and greatly facilitates the collection of flies from the drowning solution.

The efficacy of this trap-lure combination was greater than that of the commercial attractant SuzukiiTrap^®^, even after a 20 day period under laboratory conditions. The average number of *D*. *suzukii* caught in traps containing the tube device with fermenting yeast and sugar was 6.8-fold higher than observed in the SuzukiiTrap^®^ treatment on the first day of the trial, but this difference gradually decreased to 1.8-fold after a period of 20 days (Fig 3B). This reduction may be due to a gradual reduction in fermentation activity or a change in the mixture of volatiles released from the tube device or evaporation of ethanol, or a combination of these factors over time. The liberation of CO_2_ during fermentation is likely to influence the mixture of volatiles, composition and release of volatile compounds from the trap that resulted in a very high initial capture efficacy that gradually decreased over time as fermentation activity became limited by the availability of sugar substrate. In this respect the vinegar fly *D*. *melanogaster* exhibits strong avoidance behavior in response to CO_2_, but avoidance is completely abolished when CO_2_ is present with other odorants present in over-ripe fruit, yeast and beer [30].

SuzukiiTrap^®^ was used as a reference commercial lure because it captured a significantly higher number of *D*. *suzukii* than ACV alone [18, 24, 28] and was similar in efficacy, or occasionally outperformed, other yeast-sugar suspensions comprising *S*. *cerevisiae* and *Hanseniaspora uvarum* (Niehaus), when evaluated in cherry and raspberry crops [18] or blueberry [24].

In the field, it is important to consider that yeast-sugar baits used for attraction and retention in previous studies [20, 22, 24], are likely to become contaminated and undergo decomposition as many insects become trapped and drown in the lure during the trapping period. The complex communities of microorganisms associated with insects in the field, and their subsequent proliferation in the lure, could have the potential to modify the volatile profile of a lure that also serves as a drowning solution, making it less attractive for *D*. *suzukii*. This effect may be variable and unpredictable as different quantities of insects and microorganisms could enter the trap, depending on the region, crop, season and production system, among others. As such, this trap-lure combination model, in which the fermenting attractant is located in the tube device and protected with a 0.2 mm mesh, prevents insects from contaminating the yeast-sugar mixture while allowing volatiles to escape and mix with those of the ACV + ethanol drowning solution in the trap headspace.

When evaluated under field conditions in a guava orchard, this trap-lure combination was 15.5-fold more effective in trapping *D*. *suzukii* flies than ACV alone, between 4.4 and 7.3-fold more effective than SuzukiiTrap^®^, and 1.7-fold more effective than the fermenting mixture previously evaluated by Hampton et al. [20]. In commercial blackberry crops, this trap-lure combination was 6-fold more effective in catching *D*. *suzukii* flies than clear traps baited with ACV commonly used by growers in the region.

The physical features of this trap-lure combination, particularly the entrance hole diameter, avoids the capture of beneficial insects like bees, predators and parasitoids, among others. The entry of insects to the trap is almost exclusively related to other drosophilids or small flies. Together with the pest *D*. *suzukii*, other drosophilid species were trapped in orchards. In guava, *D*. *suzukii* comprised fell from 60 to 23% of the total drosophilids captured in successive trials. This increase of other drosophilid species and the lower relative prevalence of *D*. *suzukii* observed in successive trials in guava was related to the presence of fallen damaged fruit beneath guava trees that were rapidly exploited by drosophilid species other than *D*. *suzukii* [29]. However, in blackberry crops over 60% of drosophilids captured were *D*. *suzukii*. Previous field studies with different trap-lure combinations found that *D*. *suzukii* comprised less than 33% of captured drosophilids [13, 15, 22], or up to 71% in one study [12].

Drosophilids are widely attracted to fermenting products [31–34] and diversity in the capture of drosophilid species with this kind of attractants has been previously reported in studies targeted at *D*. *suzukii* [22], or the invasive species *Z*. *indianus* [35].

Interestingly, no parasitoids were captured by our 2C-Trap in orchard or greenhouse trials. Several species of parasitoids have been identified attacking *D*. *suzukii* in its native region [36] and in invaded regions, including Europe and the US [37, 38], and parasitoids were found to have been captured in ACV-baited traps in California [39]. Future studies should consider the capture of beneficial insects, including parasitoids, by trap-lure combinations targeted at *D*. *suzukii*.

In conclusion, our results have led to the development of a simple, cheap and easy to make trap which has specific features that increase its effectiveness in the field and make it more versatile than other commonly used traps. In fact, the unit cost for the 2C-Trap was less than US$0.30 including the additional device containing the sugar-yeast mixture. The trap could be used with a wide combination of attractants, with different lures for attraction and retention, which increase its effectiveness or selectivity under particular conditions of the pest, crop and season, making it a promising device for mass trapping or for use as a toxic bait station. Future studies should focus on comparing the efficacy with other commercial and handmade prototypes across a wide range of berry production greenhouses and in other crops that are highly susceptible to attack by this pest.

## Supporting information

S1 Table Pesticides sprayed on blackberry crops in greenhouses during the study. (PDF)

S1 Data Experimental data. (ZIP)
