# Supplementary material for: Improved capture of Drosophila suzukii by a trap baited with two attractants in the same device
Source: PLoS One. 2017 Nov 17;12(11):e0188350. doi: 10.1371/journal.pone.0188350 (PMC5693462; doi:10.1371/journal.pone.0188350)
Supplement: S1 Table — (PDF) [file pone.0188350.s001.pdf]

Table 1. Pesticides sprayed on blackberry crops in greenhouses during the study.

Active ingredient	Trade name	Dose ml/ha	Number of applications	Date
Chlorantraniliprole	Coragen 20 SC®	170.5	1	03 May 2017
Z-Cypermethrin	Mustang Max EW®	40	2	16 May 2017
